# Neuromorphic Event-Based 3D Pose Estimation

**DOI:** 10.3389/fnins.2015.00522

**Published:** 2016-01-22

**Authors:** David Reverter Valeiras, Garrick Orchard, Sio-Hoi Ieng, Ryad B. Benosman

**Affiliations:** ^1^Natural Vision and Computation Team, Institut de la VisionParis, France; ^2^Temasek Labs, National University of SingaporeSingapore

**Keywords:** neuromorphic vision, event-based imaging, 3D pose estimation, event-based computation, tracking

## Abstract

Pose estimation is a fundamental step in many artificial vision tasks. It consists of estimating the 3D pose of an object with respect to a camera from the object's 2D projection. Current state of the art implementations operate on images. These implementations are computationally expensive, especially for real-time applications. Scenes with fast dynamics exceeding 30–60 Hz can rarely be processed in real-time using conventional hardware. This paper presents a new method for event-based 3D object pose estimation, making full use of the high temporal resolution (1 μs) of asynchronous visual events output from a single neuromorphic camera. Given an initial estimate of the pose, each incoming event is used to update the pose by combining both 3D and 2D criteria. We show that the asynchronous high temporal resolution of the neuromorphic camera allows us to solve the problem in an incremental manner, achieving real-time performance at an update rate of several hundreds kHz on a conventional laptop. We show that the high temporal resolution of neuromorphic cameras is a key feature for performing accurate pose estimation. Experiments are provided showing the performance of the algorithm on real data, including fast moving objects, occlusions, and cases where the neuromorphic camera and the object are both in motion.

## 1. Introduction

This paper addresses the problem of 3D pose estimation of an object from the visual output of an asynchronous event-based camera if an approximate 3D model of the object is known (Lepetit and Fua, 2005). Current 3D pose estimation algorithms are designed to work on images acquired at a fixed rate by iteratively correcting errors in the focal plane until a correct estimate is found from a single image. Image acquisition is conventionally limited to the order of tens of milliseconds in real-time applications. Low frame rates usually restrict the ability to estimate robustly the pose of moving objects. Increasing the frame rate is often not a solution because the large amount of acquired data sets a limit to real-time computation. This real-time limitation is currently the bottleneck of several computer vision applications, where there is always a trade-off to find between frame rate and computational load.

A recent and evolving branch of artificial vision exploits the unique characteristics of a novel family of asynchronous frame-free vision sensors whose principle of operation is based on abstractions of the functioning of biological retinas (Delbrück et al., 2010). These event-based sensors acquire the content of scenes the changes in scenes asynchronously. Every pixel is independent and autonomously encodes visual information in its field of view into precisely timestamped events. As soon as change or motion is involved, which is the case for most machine vision applications, the universally accepted paradigm of visual frame acquisition becomes fundamentally flawed. If a camera observes a dynamic scene, no matter where the frame rate is set to, it will always be wrong. Because there is no relation whatsoever between dynamics present in a scene and the chosen frame rate controlling the pixel's data acquisition process, over-sampling or under-sampling will occur, and moreover both will usually happen at the same time. As different parts of a scene usually have different dynamic contents, a single sampling rate governing the exposure of all pixels in an imaging array will naturally fail to adequately acquire all these different simultaneously present dynamics.

Consider a natural scene with a fast moving object in front of static background, such as a pitcher throwing a baseball. When acquiring such a scene with a conventional video camera, motion blurring and displacement of the moving object between adjacent frames will result from under-sampling the fast motion of the ball, while repeatedly sampling and acquiring static background over and over again will lead to large amounts of redundant, previously known data that do not contain any new information. As a result, the scene is simultaneously under- and over-sampled. There is nothing that can be done about this sub-optimal sampling as long as all pixels of an image sensor share a common timing source that controls exposure intervals (such as a frame-clock).

Most vision algorithms, specially when dealing with dynamic input, have to deal with a mix of useless and bad quality data to deliver useful results, and continuously invest in power and resource-hungry complex processing to make up for the inadequate acquisition. This brute-force approach may however no longer be suitable in view of new vision tasks that ask for real-time scene understanding and visual processing in environments with limited power, bandwidth, and computing resources, such as mobile battery-powered devices, drones or robots.

The increasing availability and the improving quality of neuromorphic vision sensors open up the potential to introduce a shift in the methodology of acquiring and processing visual information in various demanding machine vision application (Benosman et al., 2011, 2012). As we will show, asynchronous acquisition allows us to introduce a novel computationally efficient and robust visual real-time 3D pose estimation method that relies on the accurate timing of individual pixels' response to visual stimuli. We will further show that asynchronous acquisition allows us to develop pose estimation techniques that can follow patterns at an equivalent frame rate of several kHz overcoming occlusions at the lowest computational cost. Processing can be performed on standard digital hardware and takes full advantage of the precise timing of the events.

Frame based stroboscopic acquisition induces massively redundant data and temporal gaps that make it difficult to estimate the pose of a 3D object without computationally expensive iterative optimization techniques (Chong and Zak, 2001). 3D pose estimation is a fundamental issue with various applications in machine vision and robotics such as Structure From Motion (SFM) (Snavely et al., 2007; Agarwal et al., 2011), object tracking (Drummond and Cipolla, 2002), augmented reality (Van Krevelen and Poelman, 2010) or visual servoing (Janabi-Sharifi, 2002; Janabi-Sharifi and Marey, 2010). Numerous authors have tackled finding a pose from 2D-3D correspondences. Methods range from simple approaches like DLT (Chong and Zak, 2001) to complex ones like PosIt (DeMenthon and Davis, 1995). There are two classes of techniques: iterative (DeMenthon and Davis, 1995; Kato and Billinghurst, 1999) or non-iterative (Chong and Zak, 2001; Lepetit et al., 2007). However, most techniques are based on a linear or non-linear system of equations that needs to be solved, differing mainly by the estimation techniques used to solve the pose equations and the number of parameters to be estimated.

Existing algorithms differ in speed and accuracy, some provide a fixed computation time independent of the number of points of the object (Lepetit et al., 2007). The DLT (Chong and Zak, 2001) is the simplest, slowest and weakest approach for estimating the 12 parameters in the projection matrix. However, it can be used to provide an initial estimate of the pose. PosIt (DeMenthon and Davis, 1995) is a fast method that does not use a perspective projection, but instead relies on an orthographic projection to estimate fewer parameters. The method was later extended (Oberkampf and DeMenthon, 1996) to take into account planar point clouds.

Recently, CamPoseCalib has been introduced (MIP, CAU Kiel, Germany, 2008), it is based on the Gauss-Newton method and non-linear least squares optimization (Araujo et al., 1996). Another way to solve the pose problem from point correspondences is known as the P*n*P (Perspective-*n*-Point) problem. It has been explored decades ago, readers can refer to Fischler and Bolles (1981), Lepetit et al. (2009). Other methods are based on edge correspondences (Harris, 1993; Drummond and Cipolla, 2002), or photometric information (Kollnig and Nagel, 1997).

This paper proceeds with an introduction to event-based vision sensors (Section 2.1), before describing our event-based 3D pose estimation algorithm (Section 2.2). In Section 3 we describe experiments and results obtained by our algorithm before concluding in Section 4.

## 2. Materials and methods

### 2.1. Neuromorphic silicon retina

Event-based cameras are a new class of biomimetic vision sensors that, unlike conventional frame-based cameras, are not driven by artificially created clock signals. Instead, they transmit information about the visual scene in an asynchronous manner, just like their biological counterparts. One of the first attempts of incorporating the functionalities of the retina in a silicon chip is the work of Mahowald (1992) in the late eighties. Since then, the most interesting achievements in neuromorphic imagers has been the development of activity-driven sensing. Event-based vision sensors output compressed digital data in the form of events, removing redundancy, reducing latency, and increasing dynamic range when compared with conventional cameras. A complete review of the history and existing sensors can be found in Delbrück et al. (2010). The Asynchronous Time-based Image Sensor (ATIS; Posch et al., 2011) used in this work is an Address-Event Representation (AER; Boahen, 2000) silicon retina with 304 × 240 pixel resolution.

**Figure 1:**
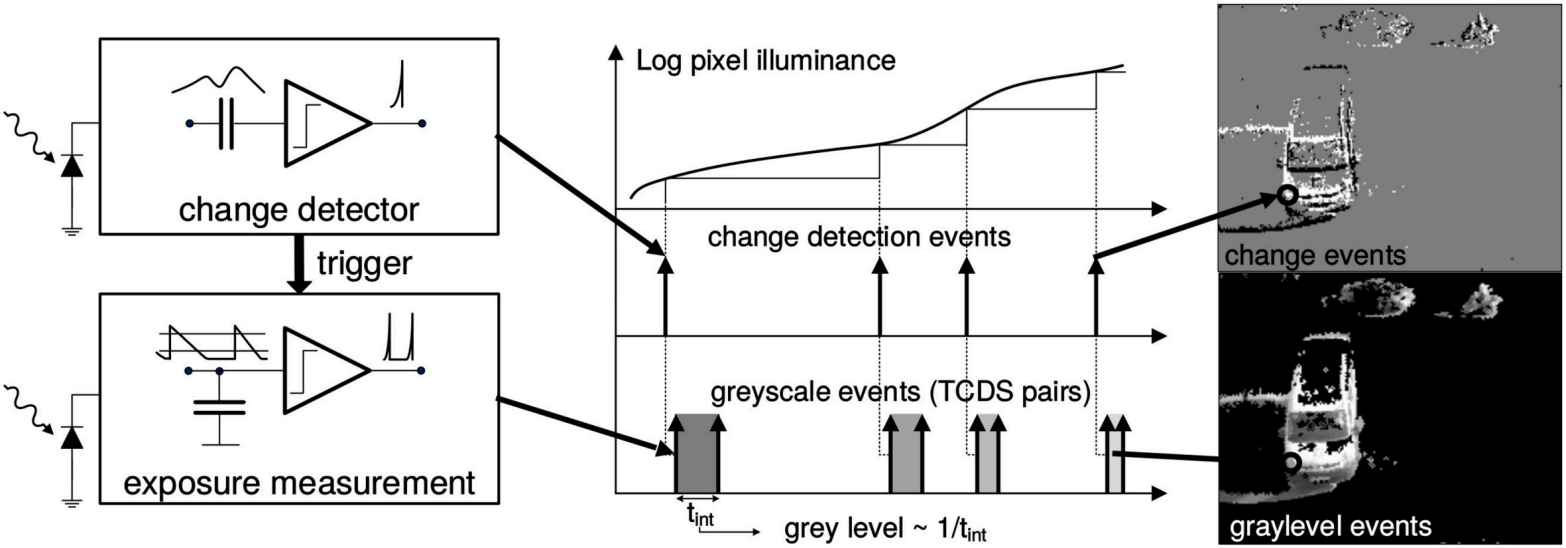
**Functional diagram of an ATIS pixel (Posch et al., 2011)**. Two types of asynchronous events, encoding change **(top)** and illuminance **(bottom)** information, are generated and transmitted individually by each pixel in the imaging array when a change is detected in the scene. The bottom right image only shows grayscale of pixels for which illuminance has recently been measured. Black pixels indicate locations where illuminance has not been measured recently.

The ATIS output consists of asynchronous address-events that signal scene illuminance changes at the times they occur. Each pixel is independent and detects changes in log intensity larger than a threshold since the last emitted event (typically 15% contrast). As shown in Figure 1, when the change in log intensity exceeds a set threshold an ON or OFF event is generated by the pixel, depending on whether the log intensity increased or decreased. Immediately after, the measurement of an exposure/grayscale value is initiated, which encodes the absolute pixel illuminance into the timing of asynchronous event pulses, more precisely into inter-event intervals. The advantage of such a sensor over conventional clocked cameras is that only moving objects produce data. Thus, the amount of redundant information and the load of post-processing are reduced, making this technology particularly well-suited for high-speed tracking applications. Additionally, the timing of events can be conveyed with very low latency and accurate temporal resolution of 1 μ s. Consequently, the equivalent frame rate is typically several kHz. The encoding of log intensity of light change implements a form of local gain adaptation which allows them to work over scene illuminations that range from 2 lux to over 100 klux. When events are sent out, they are timestamped using off-chip digital components and then transmitted to a computer using a standard USB connection.

The present algorithm estimates 3D pose using only change detector events. The corresponding stream of events can be mathematically described in the following way: let $e_{k}={\left(u_{k}^{T},t_{k},p_{k}\right)}^{T}$ be a quadruplet describing an event occurring at time *t*_*k*_ at the position $u_{k}={\left(x_{k},y_{k}\right)}^{T}$ on the focal plane. The two possible values for the polarity, *p*_*k*_, are 1 or −1, depending on whether a positive or negative change of illuminance has been detected.

### 2.2. Event-based 3D pose estimation

In the first two subsections below we formulate the 3D pose estimation problem and describe the notation we use for 3D rotations. In the subsequent two subsections we describe how we match incoming visual events to edge projections on the focal plane, and how we then match these events to the 3D locations of points on the object. Finally, in the fifth subsection below we describe how we update our model of the object's 3D pose using these event-based correspondences.

#### 2.2.1. Problem formulation

**Figure 2:**
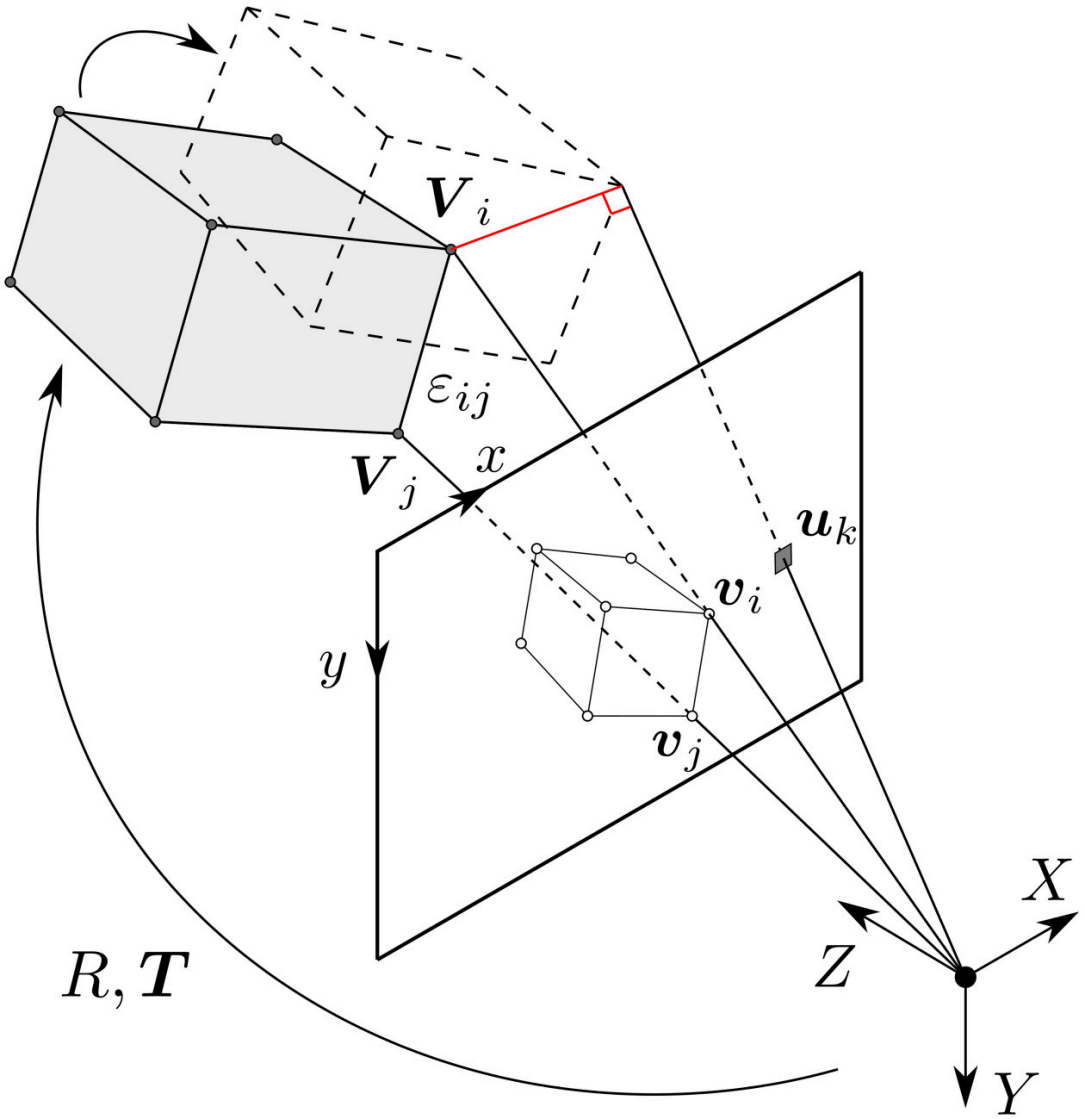
**The object is described as a set of vertices {***V**_***i***_*}, whose corresponding projections on the focal plane are denoted as {***v**_***i***_*}**. An edge defined by vertices ***V***_*i*_, ***V***_*j*_ is noted ε_*ij*_. If an event *e*_*k*_ has been generated by a point ***V***_*i*_ of the model, then ***V***_*i*_ must lie on the line of sight of the event, which is the line passing through the camera center and the position of the event on the focal plane ***u***_*k*_. When that happens, the projection of the point ***v***_*i*_ is guaranteed to be aligned with the event.

Let us consider a moving rigid object observed by a calibrated event-based camera. The movement of the object generates a stream of events on the focal plane of the camera. Attached to this object is a frame of reference, known as the *object-centered reference frame*, whose origin we denote as ***V***_0_. The pinhole projection maps 3D points ***V*** expressed in the object-centered reference frame into υ on the camera's focal plane (see Figure 2), according to the relation:

(1) $$\begin{array}{l} \left(\begin{array}{c} \upsilon \\ 1 \end{array}\right)\sim K(R\;\;T)\left(\begin{array}{c} V\\ 1 \end{array}\right). \end{array}$$

Here, *K* is the 3 × 3 matrix defining the camera's intrinsic parameters—obtained through a prior calibration procedure— while ***T*** ∈ ℝ^3^ and *R* ∈ *SO*(3) are the extrinsic parameters. The sign ~ indicates that the equality is defined up to a scale (Hartley and Zisserman, 2003). (***T**, R*) are also referred to as the relative pose between the object and the camera (Murray et al., 1994). As the object moves, it is only the pose which changes and needs to be estimated.

An estimation of the pose can be found by minimizing the orthogonal projection errors on the line of sight for each 3D point, as illustrated by Figure 2. Thus, we minimize a cost function directly on the 3D structure rather than computing it on the image plane (Lu et al., 2000). The advantage of this approach is that a correct match of the 3D points leads to a correct 2D projection on the focal plane, but the reverse is not necessarily true.

The high temporal resolution of the camera allows us to acquire a smooth trajectory of the moving object. We can then consider each event generated by the moving object as relatively close to the previous position of the object. Since the event-based camera detects temporal contours, all moving objects can be represented by a set of vertices and edges. We can then set the following convention: let {**V**_*i*_} be the set of 3D points defining an object. These 3D points are vertices and their projections onto the retina focal plane are noted as υ_*i*_. The edge defined by vertices ***V***_*i*_, ***V***_*j*_ is noted as ε_*ij*_. Figure 2 shows a general illustration of the problem.

Using the usual computer graphics conventions (O'Rourke, 1998; Botsch et al., 2010), an object is described as a polygon mesh. This means that all the *faces* of the model are simple polygons, triangles being the standard choice. The boundaries of a *face* are defined by its *edges*.

#### 2.2.2. Rotation formalisms

A convenient parametrization for the rotation is to use unit quaternions (Murray et al., 1994). A quaternion is a 4-tuple, providing a more efficient and less memory intensive method of representing rotations compared to rotation matrices. It can be easily used to compose any arbitrary sequence of rotations. For example, a rotation of angle ϕ about rotation axis ***r*** is represented by a quaternion *q* satisfying:

(2) $$\begin{array}{l} q\left(\phi ,r\right)=\cos\frac{\phi }{2}+r\sin\frac{\phi }{2}, \end{array}$$

where ***r*** is a unit vector. In what follows, we will use the quaternion parametrization for rotations.

When trying to visualize rotations, we will also use the axis-angle representation, defined as the rotation vector ϕ***r***.

#### 2.2.3. 2D edge selection

**Figure 3:**
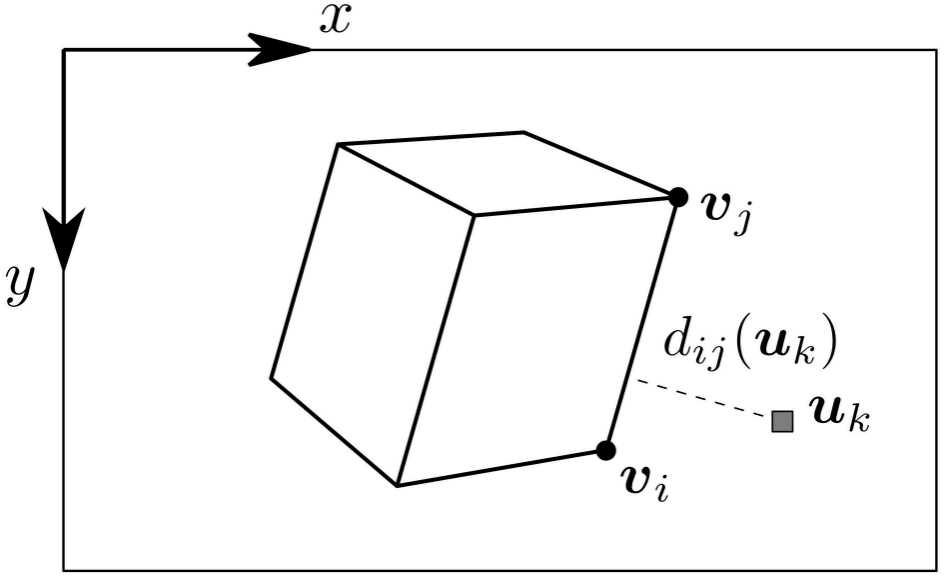
**Edge selection for an event ***e***_***k***_ occurring at ***u***_***k***_**. The distance of ***u***_*k*_ to each visible edge ε_*ij*_ is computed as *d*_*ij*_(***u***_*k*_), the euclidean distance between ***u***_*k*_ and the segment defined by the projected edge [***v***_*i*_, ***v***_*j*_].

The model of the tracked object and its initial pose are assumed to be known. This allows us to virtually project the model onto the focal plane as a set of edges. For each incoming event *e*_*k*_ occurring at position $u_{k}={\left(x_{k},y_{k}\right)}^{T}$ on the image plane, we are looking for the closest visible edge. Thus, for every visible edge ε_*ij*_, projected on the focal plane as the segment [υ_*i*_, υ_*j*_], we compute *d*_*ij*_(***u***_*k*_), the euclidean distance from ***u***_*k*_ to [υ_*i*_, υ_*j*_] (see Figure 3). To compute this distance, ***u***_*k*_ is projected onto the line defined by [υ_*i*_, υ_*j*_]. If this projection falls inside of the segment [υ_*i*_, υ_*j*_], then the distance is given by the generic expression:

(3) $$\begin{array}{l} d_{ij}\left(u_{k}\right)=\frac{∥\left(u_{k}-\upsilon_{i}\right)\times \left(\upsilon_{j}-\upsilon_{i}\right)∥}{∥\upsilon_{j}-\upsilon_{i}∥,} \end{array}$$

where × is the cross product. If the projection is not inside [υ_*i*_, υ_*j*_], then *d*_*ij*_(***u***_*k*_) is set to be equal to the distance between ***u***_*k*_ and the closest endpoint.

We set a maximum allowed distance for the event to be assigned to an edge as *d*_*max*_. The edge to which the event is assigned to is ε_*nm*_ such that:

(4) $$d_{nm}(u_{k})=\;\underset{i,j}{\min}d_{ij}(u_{k}),$$

assuming *d*_*nm*_(***u***_*k*_) ≤ *d*_*max*_, otherwise the event is considered as noise and discarded.

**Remark 1**: In complex scenarios, the 2D matching step can be further strengthened by applying more refined criteria. We implement a 2D matching based on Gabor events, which are oriented events generated by events lying on a line (Orchard et al., 2015). When the 2D matching is performed using this technique, a Gabor event will only be assigned to a visible edge if the angle of the event and the angle formed by the edge are close enough. An example of application of this method will be shown in the experiments, where pose estimation is performed even with partial occlusions and egomotion of the camera.

**Remark 2**: This section assumes that the visibility of the edges is known. This is done via a *Hidden Line Removal* algorithm (Glaeser, 1994) applied for each new pose of the model.

#### 2.2.4. 3D matching

Once ε_*nm*_ is determined, we need to look for the point on the edge that has generated the event. The high temporal resolution of the sensor allows us to set this point as the closest to the line of sight of the incoming event. Performing this matching between an incoming event in the focal plane and a physical point on the object allows to overcome issues that appear when computation is performed directly in the focal plane. The perspective projection on the focal plane is neither preserving distances nor angles, i.e., the closest point on the edge in the focal plane is not necessarily the closest 3D point of the object.

The camera calibration parameters allow us to map each event at pixel ***u***_*k*_ to a line of sight passing through the camera's center. The 3D matching problem is then equivalent to a search for the smallest distance between any two points lying on the object's edge and the line of sight.

**Figure 4:**
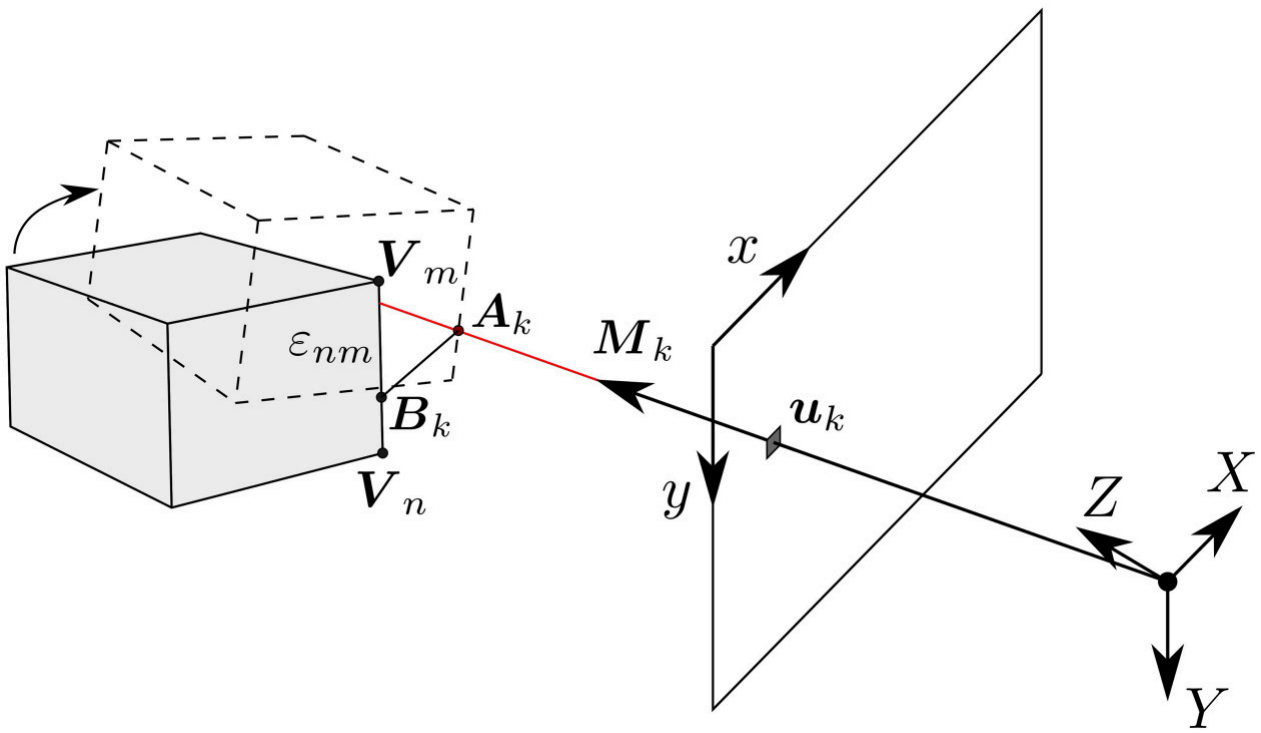
**Geometry of the 3D matching problem: an event ***e***_***k***_ at position $u_{k}={\left(x_{k},y_{k}\right)}^{T}$ is generated by a change of luminosity in the line of sight passing through the event, defined by the vector *M*_*k*_**. ***A***_*k*_ is a point on the line of sight and ***B***_*k*_ a point on the edge ε_*nm*_, such that the minimum distance between these two lines is reached. Finding ***A***_*k*_ and ***B***_*k*_ is the objective of the 3D matching step.

As shown in Figure 4, let ***A***_*k*_ be a point on the line of sight of an incoming event *e*_*k*_ located at ***u***_*k*_ in the focal plane. Let ***B***_*k*_ be a point on the edge ε_*nm*_ that has been computed as being at a minimal distance from the line of sight passing through ***u***_*k*_. We can assume *e*_*k*_ to be generated by a 3D point on the moving object at the location ***A***_*k*_, that was at ***B***_*k*_ before *e*_*k*_ occurred. This hypothesis is reasonable as due to the high temporal resolution events are generated by small motions. Finding ***A***_*k*_ and ***B***_*k*_ is the scope of the 3D matching step.

Let ***M***_*k*_ be the vector defining the line of sight of *e*_*k*_, it can be obtained as:

(5) $$\begin{array}{l} M_{k}=K^{-1}\left(\begin{array}{c} u_{k}\\ 1 \end{array}\right). \end{array}$$

***A***_*k*_ and ***B***_*k*_ can therefore be expressed as:

(6) $$\begin{array}{l} A_{k}=\alpha_{1}M_{k} \end{array}$$

(7) $$\begin{array}{l} B_{k}=V_{n}+\alpha_{2}\left(V_{m}-V_{n}\right), \end{array}$$

where α_1_ and α_2_ are two real valued parameters.

Let ε_*nm*_ = ***V***_*m*_ − ***V***_*n*_, we are looking for solutions such that (***A***_*k*_ − ***B***_*k*_) is perpendicular to both ε_*nm*_ and ***M***_*k*_. Hence, we obtain the following equation:

(8) $$\begin{array}{l} \left(\begin{array}{c} -M_{k}^{T}M_{k}M_{k}^{T}\varepsilon_{nm}\\ -M_{k}^{T}\varepsilon_{nm}\varepsilon_{nm}^{T}\varepsilon_{nm} \end{array}\right)\left(\begin{array}{c} \alpha_{1}\\ \alpha_{2} \end{array}\right)=\left(\begin{array}{c} -V_{n}^{T}M_{k}\\ -V_{n}^{T}\varepsilon_{nm} \end{array}\right). \end{array}$$

Solving this equation for α_1_ and α_2_ provides both ***A***_*k*_ and ***B***_*k*_. The solution to this system is discussed in the Appendix.

We also set a maximum 3D distance between ***A***_*k*_ and ***B***_*k*_, denoted *D*_*max*_. If the distance between ***A***_*k*_ and ***B***_*k*_ is larger than this value we discard the event.

#### 2.2.5. Rigid motion estimation

Knowing ***B***_*k*_ and ***A***_*k*_ allows us to estimate the rigid motion that transforms ***B***_*k*_ into ***A***_*k*_. We define two strategies: the direct estimation of the required transformation for every incoming event and the computation using an estimation of the velocity.

#### Direct transformation

The rigid motion is composed of a translation **Δ***T_k_* and a rotation Δ*q*_*k*_ around ***V***_0_, the origin of the object-centered reference frame.

Let us define the scaling factor λ_*T*_ such that **Δ***T_k_* is related to the vector ***A***_*k*_ − ***B***_*k*_ as:

(9) $$\begin{array}{l} {\Delta T}_{k}=\left(\begin{array}{ccc} 1 & 0 & 0\\ 0 & 1 & 0\\ 0 & 0 & m \end{array}\right)\lambda_{T}\left(A_{k}-B_{k}\right), \end{array}$$

where (***A***_*k*_ − ***B***_*k*_) is the translation that makes ***B***_*k*_ coincide with ***A***_*k*_. Here, *m* is a multiplier that allows us to set the scaling factor independently for the *Z* axis. The need for this extra degree of freedom can be justified because changes in the depth of the object will only become apparent through changes in the *x* or *y* position of the events on the image plane. Consequently, the system does not react in the same way to changes in depth as it does to changes in the *X* or *Y* position, resulting in a different latency for the *Z* axis. *m* is then a tuning factor that will be set experimentally.

The rotation around ***V***_0_ is given by a unit quaternion Δ*q*_*k*_ of the form:

(10) $$\Delta q_{k}(\lambda_{\theta }\theta_{k},h_{k})=\cos\left(\frac{\lambda_{\theta }\theta_{k}}{2}\right)+h_{k}\sin\left(\frac{\lambda_{\theta }\theta_{k}}{2}\right),$$

where ***h***_*k*_ is a unit vector collinear to the axis of rotation and λ_θ_θ_*k*_ is equal to the rotation angle, that we conveniently define as a product between a scaling factor λ_θ_ and the angle θ_*k*_ defined below.

**Figure 5:**
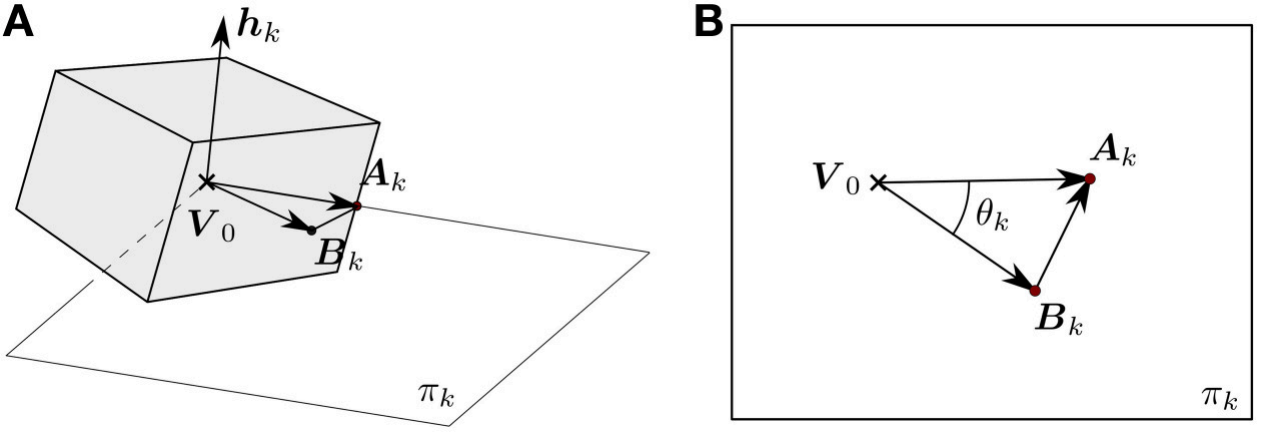
**(A)** π_*k*_ represents the plane defined by ***A***_*k*_, ***B***_*k*_ and the origin of the object-centered reference frame ***V***_0_. The desired rotation is contained in this plane, and thus the rotation axis ***h***_*k*_ is normal to it. **(B)** Normal view to π_*k*_. Both (***B***_*k*_ − ***V***_0_) and (***A***_*k*_ − ***V***_0_) are contained in this plane, and thus their cross product gives us the axis of rotation. The angle θ_*k*_ between these two vectors is equal to the rotation angle that makes ***B***_*k*_ and ***A***_*k*_ coincide.

If π_*k*_ is the plane passing through ***B***_*k*_, ***A***_*k*_ and ***V***_0_ (see Figure 5A) such that ***h***_*k*_ is the normal, then ***h***_*k*_ can be computed as:

(11) $$\begin{array}{l} h_{k}=\frac{\left(B_{k}-V_{0}\right)\times \left(A_{k}-V_{0}\right)}{∥\left(B_{k}-V_{0}\right)\times \left(A_{k}-V_{0}\right)∥}. \end{array}$$

We define θ_*k*_ as the angle between (***B***_*k*_ − ***V***_0_) and (***A***_*k*_ − ***V***_0_), as shown in Figure 5B.

(12) $$\theta_{k}=\tan^{-1}\left(\frac{\left\|(B_{k}-V_{0})\times (A_{k}-V_{0})\right\|}{{\left(B_{k}-V_{0}\right)}^{T}(A_{k}-V_{0})}\right).$$

In the case of ***A***_*k*_, ***B***_*k*_ and ***V***_0_ alignment If ***A***_*k*_, ***B***_*k*_ and ***V***_0_ are aligned, ***h***_*k*_ is undefined. This happens when no rotation is applied or when the rotation angle is equal to π. This last case is unlikely to occur because of the small motion assumption.

Finally, the pose of the model is updated incrementally according to:

(13) $$\begin{array}{l} T_{k}=T_{k-1}+{\Delta T}_{k} \end{array}$$

(14) $$\begin{array}{l} q_{k}=\Delta q_{k}q_{k-1}. \end{array}$$

For the rest of the paper, this described procedure will be referred to as the *direct transformation* strategy.

**Remark 1:** Once the pose is updated, the next step is to update the transformation between the object and the camera. This is a computationally expensive process that requires transforming the 3D points, projecting them onto the image plane and applying the hidden-line removal algorithm. Consequently, in order to increase the performance of the system, we do not apply the transformation for every incoming event, but every *N* events. *N* is experimentally chosen, and its effect on the algorithm discussed in the experiments section.

**Remark 2:** λ_*T*_ and λ_θ_ are set experimentally, and they should always be equal or smaller than one. When they are smaller than one, we do not fully transform the model so that ***B***_*k*_ matches ***A***_*k*_ for every event. Instead, we apply a small displacement for each incoming event. Here, it is important to keep in mind that a moving edge generates more than one event. This number and the frequency of events are proportional to the local contrast.

#### Velocity estimation

We make an additional hypothesis on the motion smoothness which assumes the velocity of the object does not change abruptly. This hypothesis allows us to update the velocity only after every *N* events. Due to the high temporal resolution and the asynchronous nature of the neuromorphic camera, we consider this to be, in general, a reasonable assumption.

For an incoming event *e*_*k*_, let ${\hat{\Delta T}}_{k}$ be the cumulative translation of the estimates from the last *N* events:

(15) $$\begin{array}{l} {\hat{\Delta T}}_{k}=\overset{k}{\underset{i=k-N}{\sum }}{\Delta T}_{i}, \end{array}$$

where **Δ***T_i_* is equal to the translation for the *ith* event, computed using (9) with λ_*T*_ = 1. Let us note that here, **Δ***T_i_* are not displacements to be applied to the model. Instead, we are using them to compute the cumulative translation for the last *N* events, that we will later use to estimate the mean linear velocity during that period. This fact justifies the choice of making λ_*T*_ = 1.

Analogously, let ${\hat{\Delta q}}_{k}({\hat{\theta }}_{k},{\hat{h}}_{k})$ be the quaternion of the resulting rotation associated with the last *N* events:

(16) $$\begin{array}{l} {\hat{\Delta q}}_{k}({\hat{\theta }}_{k},{\hat{h}}_{k})=\prod_{i=k-N}^{k}\Delta q_{i}(\theta_{i},h_{i}), \end{array}$$

where the quaternions Δ*q*_*i*_ are computed using (10) with λ_θ_ = 1, for the same reason as above.

From these cumulative translation and rotation, we define ν_*k*_ and ω_*k*_, the mean linear and angular velocities for the last *N* events:

(17) $$\begin{array}{l} {\bar{\nu }}_{k}=\frac{{\hat{\Delta T}}_{k}}{N\Delta t}, \end{array}$$

and

(18) $$\begin{array}{l} {\bar{\omega }}_{k}=\frac{{\hat{\theta }}_{k}}{N\Delta t}{\hat{h}}_{k}, \end{array}$$

where Δ*t* = *t*_*k*_ − *t*_*k* − *N*_.

Equations (17) and (18) have these forms because moving edges generate a certain number of events with the same timestamp, and the estimated pose is updated every *N* events. We can then consider the last *N* events to correspond to the same small motion. Consequently, the mean linear velocity ${\bar{\nu }}_{k}$ is computed as the mean displacement ${\hat{\Delta T}}_{k}∕N$ over the corresponding time interval Δ*t*. The same explanation holds for $\bar{\omega_{k}}$.

The velocities are finally updated every *N* events according to the following expressions:

(19) $$\begin{array}{l} \nu_{k}=\left(1-\lambda_{\nu }\right)\nu_{k-N}+\lambda_{\nu }{\bar{\nu }}_{k}, \end{array}$$

(20) $$\begin{array}{l} \omega_{k}=\left(1-\lambda_{\omega }\right)\omega_{k-N}+\lambda_{\omega }{\bar{\omega }}_{k}, \end{array}$$

where λ_ν_ and λ_ω_ are update factors, that will be set experimentally. Finally, the translation estimated for the model is computed as:

(21) $$\begin{array}{l} {\Delta T}_{k}=\Delta t\nu_{k}. \end{array}$$

and the rotation is deduced from the angular velocity vector with the axis being ω_*k*_∕∥ω_*k*_∥ and the angle Δ*t*∥ω_*k*_∥. This is represented by the unit quaternion Δ*q*_*k*_:

(22) $$\begin{array}{l} \Delta q_{k}\left(\Delta t∥\omega_{k}∥,\frac{\omega_{k}}{∥\omega_{k}∥}\right). \end{array}$$

Next, we update the pose of the model, which is only updated every *N* events when applying this strategy:

(23) $$\begin{array}{l} T_{k}=T_{k-N}+{\Delta T}_{k} \end{array}$$

(24) $$\begin{array}{l} q_{k}=\Delta q_{k}q_{k-N}. \end{array}$$

We will refer to this way of computing the transformation as the *velocity estimation* strategy. The general algorithm for both methods is given below (Algorithm 1).

**Algorithm 1:** Event-Based 3D pose estimation algorithm

**Require:** $e_{k}{\left(u_{k}^{T},t_{k},p_{k}\right)}^{T}\;\forall k>0$
**Ensure:** ***T**, q*
Initialize the parameters
Select the method for the rigid motion estimation
**for** every incoming event $e_{k}={\left(u_{k}^{T},t_{k},p_{k}\right)}^{T}$ **do**
**for** every visible edge ε_*ij*_ **do**
Compute the distance *d*_*ij*_(***u***_*k*_) between ***u***_*k*_ and [***v***_*i*_, ***v***_*j*_]
**end for**
*d*_*nm*_(***u***_*k*_) ← min(*d*_*ij*_(***u***_***k***_))
**if** *d*_*nm*_(***u***_*k*_) ≤ *d*_*max*_ **then**
Solve (8) in α_1_ and α_2_
Compute ***A***_*k*_ and ***B***_*k*_ using (6) and (7)
**if** ||***A***_*k*_ − ***B***_*k*_|| ≤ *D*_*max*_ **then**
Compute **Δ***T_k_* and Δ*q*_*k*_ using (9) and (10)
**if** method = direct transformation **then**
Update ***T*** and *q* using (13) and (14)
**else**
Update $\hat{\Delta T}$ and $\hat{\Delta q}$ using (15) and (16)
**end if**
**end if**
**end if**
**for** each *N* consecutive events **do**
**if** method = velocity estimation **then**
Update **ν**_*k*_ and ω_*k*_ using (19) and (20)
Compute **Δ***T_k_* and Δ*q*_*k*_ using (21) and (22)
Update ***T*** and *q* using (23) and (24)
**end if**
Apply the transformation to the model
**end for**
**end for**

## 3. Results

In this section we present experiments to test 3D pose estimation on real data^1^. The first two experiments estimate the pose of a moving icosahedron and house model while viewed by a static event-based sensor. In Section 3.3 we estimate the pose of the icosahedron from the view of a moving event-based sensor in a scene containing multiple moving objects. In Section 3.4 we estimate the pose of the icosahedron under high rotational velocity (mounted on a motor). Finally, in Section 3.5 and Section 3.6 we investigate how temporal resolution affects pose estimation accuracy, and how implementation parameters affect the time required for computation.

In what follows, we will denote the ground truth as {***T***, *q*} and the estimated pose as {T^*^, q^*^}.

**Figure 6:**
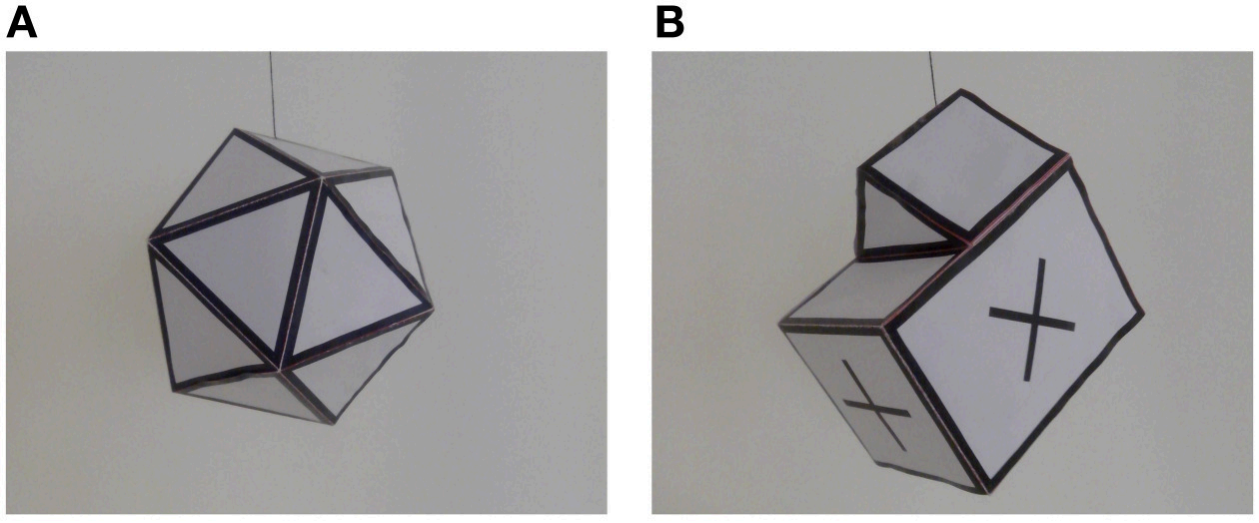
**Real objects used in the experiments**. ***(*A)** White icosahedron with black edges, used in the first experiment. ***(*B)** Non-convex model of a house with cross markers on its faces, used in the second experiment.

The algorithm is implemented in C++ and tested in recordings of an icosahedron—shown in Figure 6A—and the model of a house—Figure 6B—freely evolving in the 3D space. We set the following metrics on ℝ^3^ and *SO*(3):

- The absolute error in linear translation is given by the norm of the difference between T^*^ and ***T***. For a given recording, let $\bar{T}=\frac{1}{K}\overset{K}{\underset{k=1}{\sum }}T_{k}$ be the mean displacement of the object, where *K* is the total number of events. We define ξ_*T*_ the relative error as:
  (25) $$\begin{array}{l} \xi_{T}=\frac{∥T^{*}-T∥}{∥\bar{T}∥}. \end{array}$$
- For the rotation, the error is defined with the distance *d* between two unit quaternions *q* and q^*^:
  (26) $$\begin{array}{l} d\left(q,q^{*}\right)=min\left\{∥q-q^{*}∥,∥q+q^{*}∥\right\}, \end{array}$$
  which is proven to be a more suitable metric for *SO*(3), the space spanned by 3D rotations (Huynh, 2009). It takes values in the range $\left[0,\sqrt{2}\right]$. Thus, let ξ_*q*_ be the relative rotation error:
  (27) $$\begin{array}{l} \xi_{q}=\frac{d\left(q,q^{*}\right)}{\sqrt{2}}. \end{array}$$

The algorithm provides an instantaneous value of the errors for each incoming event. In order to characterize its accuracy, we will consider $\bar{\xi_{T}}$ and $\bar{\xi_{q}}$, the temporal mean of the errors for the whole duration of a given recording.

### 3.1. Icosahedron

The icosahedron shown in Figure 6A is recorded by an ATIS sensor for 25 s while freely rotating and moving. The 3D model is a mesh of 12 vertices and 20 triangular faces.

The ground truth is built from frames output from the event-based camera. We have manually selected the image position of the visible vertices every 100 ms and applied the OpenCV implementation of the EP*n*P (Efficient Perspective-*n*-Point) algorithm (Lepetit et al., 2009) to estimate the pose. In Lepetit et al. (2009), the authors test the robustness of their algorithm to gaussian noise perturbations on the focal plane. It is important to outline that this is a theoretical disturbance model. They are not assessing their algorithm's performance with real noisy data. Based on their noise model results, we can give an order of magnitude of the ground truth accuracy. Assuming that the manual annotation of the vertices of the icosahedron has at least 2 pixels precision, we can read the pose error from the error curves (Figure 5 in Lepetit et al., 2009), that is at most 2 %.

The intermediate positions are obtained by linear interpolation, and the intermediate rotations using Slerp (spherical linear interpolation Shoemake, 1985). From the ground truth we compute the model's linear velocity **ν** and the angular velocity ω. In this recording, the linear speed ∥**ν**∥ reaches a maximum of 644.5 mm/s, while the angular speed ∥ω∥ starts with a maximum of 2.18 revolutions per second at the beginning of the recording and then continuously decreases.

After several trials, the thresholds are set experimentally to values giving stable and repeatable pose estimations. These are: *d*_*max*_ = 20 pixels and *D*_*max*_ = 10 mm. The remaining tuning parameters are experimentally chosen for each experiment as the ones giving the smallest sum of the mean relative estimation errors $\bar{\xi_{T}}$ and $\bar{\xi_{q}}$. The update factors λ_*T*_, λ_θ_, λ_ν_, λ_ω_ are always taken between 0.001 and 0.4, a large range in which the algorithm has proven to yield stable results.

**Figure 7:**
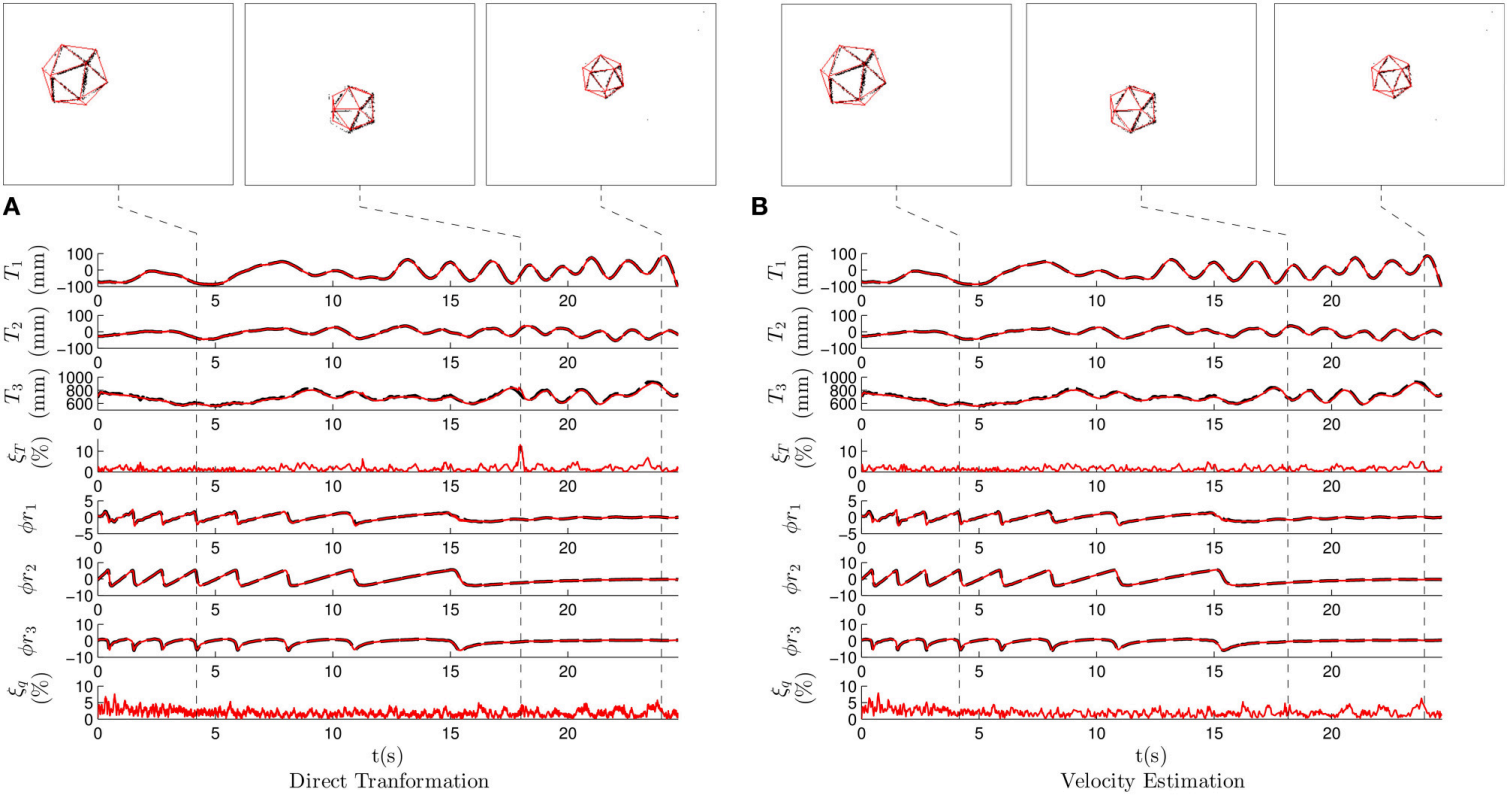
**Results for the first experiment, where we recorded an icosahedron freely evolving in the 3D space**. *T*_1_, *T*_2_, and *T*_3_ are the components of the translation vector ***T***, and ϕ*r*_1_, ϕ*r*_2_, ϕ*r*_3_ the components of the axis-angle representation of the rotation ϕ***r***. The dashed lines represent ground truth, while the solid curves represent estimated pose. The snapshots on the top show the state of the system in some characteristic moments, with the estimation made by the algorithm printed over the events. **(A)** Results when applying the *direct transformation* strategy, with λ_*T*_ = 0.4, λ_θ_ = 0.2, *N* = 1 and *m* = 2. **(B)** Results when applying the *velocity estimation* strategy with λ_ν_ = 0.05, λ_θ_ = 0.006, *N* = 5 and *m* = 10. We verify that the estimation and the ground truth are coincidental most of the time, allowing us to conclude that pose estimation is in general correctly performed.

Figure 7A shows the results when applying the *direct transformation* strategy with λ_*T*_ = 0.4, λ_θ_ = 0.2, *N* = 1 and *m* = 2. We show the translation vector ***T*** as well as the rotation vector ϕ***r***. Plain curves, representing estimation results, are superimposed with dashed lines indicating the ground truth. Snapshots, showing the state of the system at interesting instants are shown. They provide the projection of the shape on the focal plane using the estimated pose.

We verify that plain and dashed lines (representing estimated and ground truth poses respectively) coincide most of the time, showing that the pose estimation is in general correctly performed. Experiments provide the following mean estimation errors: $\bar{\xi_{T}}=1.48\%$ for the translation, and $\bar{\xi_{q}}=1.96\%$ for the rotation. Instantaneous errors reach a local maximum, as a consequence of the large values chosen for λ_*T*_ and λ_θ_. These parameters being gains, large values imply an oscillatory behavior around the correct pose parameters. We include in Figure 7A a snapshot showing the state of the system at this instant, where we observe that the estimation is slightly displaced from the true pose. However, even when considering this local maximum, the estimation errors remain below 15%. The system is always capable of recovering the correct pose.

Figure 7B shows the results when applying the *velocity estimation* strategy with λ_ν_ = 0.05, λ_θ_ = 0.006, *N* = 5 and *m* = 10. The estimation of the pose is accurate: $\bar{\xi_{T}}=1.40$ and 2.04%. The mean errors obtained are very similar to the ones produced in the case of the *direct transformation* strategy. However, when we analyze the instantaneous errors, we do not observe a large local maxima as in the previous case. The *velocity estimation* strategy assumes that the velocity of the object does not change abruptly, and consequently, the estimated motion is smoother. This constitutes the main advantage of the velocity strategy over the previous one. This will be further outlined in the following experiment.

The output of the algorithm for this experiment can be seen in Supplementary Video 1, where the results produced by both strategies are shown.

### 3.2. House

This experiment tests the accuracy of the algorithm using a more complex model of a house shown in Figure 6B. The object is recorded for 20 s while freely rotating and moving in front of the camera. The 3D model is composed of 12 vertices and 20 triangular faces. We compute velocities from the ground truth obtained from generated frames as was done with the icosahedron. In this case, the linear speed reaches a maximum of 537.4 mm/s, while the angular speed starts with a maximum of 1.24 revolutions per second at the beginning of the experiment and then continuously decreases.

**Figure 8:**
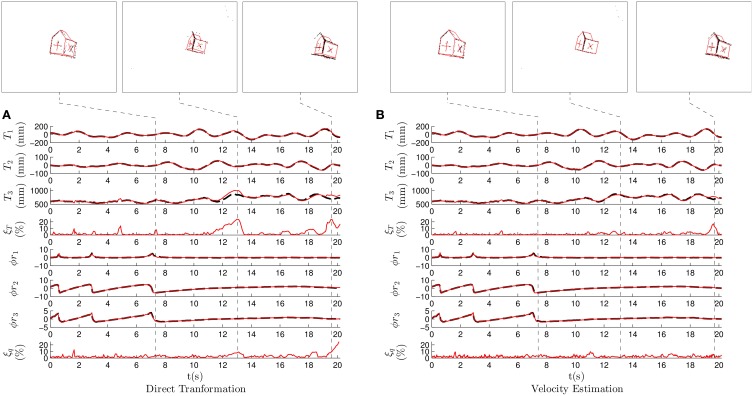
**Results for the second experiment, where we recorded a non-convex model of a house freely evolving in the 3D space**. **(A)** Translation and rotation results when applying the *direct transformation* strategy. **(B)** Translation and rotation results when applying the *velocity estimation* strategy.

As in the previous case, we experimentally choose the set of parameters that produces the minimum sum of errors. Figure 8A shows the results when applying the *direct transformation* strategy with λ_*T*_ = 0.2, λ_θ_ = 0.05, *m* = 1 and *N* = 10. We verify that there is a coherence between the ground truth and the estimated pose showing that the pose estimation is in general correctly estimated. However, in this case we observe a larger local maxima reaching values as high as 20%. These local maxima degrade the overall performance, they provide the following values for the mean estimation errors: $\bar{\xi_{T}}=3.12\%$ for the translation and $\bar{\xi_{q}}=2.62\%$ for the rotation, higher than in the previous case. Nevertheless, the system is always capable of recovering the correct pose after these maxima, and the mean estimation errors remain acceptable.

In this recording, local maxima mostly occur because of the algorithm mistakenly interpreting the cross markers as edges or viceversa. This usually happens when a given face is almost lateral with respect to the camera. In that case, it provides the projection of these lines very close to each other. The negative effect of these ambiguous poses is difficult to mitigate when applying this strategy.

Figure 8B shows the results when the *velocity estimation* strategy is applied with λ_ν_ = 0.4, λ_ω_ = 0.0125, *m* = 4 and *N* = 5. As in the previous experiment, we verify that the effect of the local maxima is reduced when applying this strategy. This results in the following errors: $\bar{\xi_{T}}=1.53\%$ and $\bar{\xi_{q}}=2.27\%$, clearly outperforming the *direct transformation* strategy. We verify that in the case of complex objects and ambiguous poses, using an estimation of the velocity provides more robust results. In this case, the small value for λ_ω_ makes the angular velocity very stable, preventing the estimation from rapidly switching from one pose to another. It also reduces the negative effect of the ambiguous poses.

Pose estimation is accurate in both the presented cases, but the *velocity estimation* strategy provides more stable results. Results produced by both strategies when treating this recording are shown in Supplementary Video 2.

### 3.3. 2D matching using gabor events

In this experiment we test pose estimation in a more complex scenario, with egomotion of the camera and partial occlusions of the object, using Gabor events for the 2D matching step. A hand-held icosahedron is recorded for 20 s while the camera moves. Ground truth is obtained from reconstructed frames as in the previous experiments.

The parameters for the Gabor events' generation process are set as in Orchard et al. (2015), and the maximum angular distance for an event to be assigned to an edge is set as 0.174 rad (obtained as $\frac{\pi }{1.5\times 12}$, where 12 is the number of different orientations that the Gabor events can take). The tuning parameters are experimentally chosen as in previous experiments.

**Figure 9:**
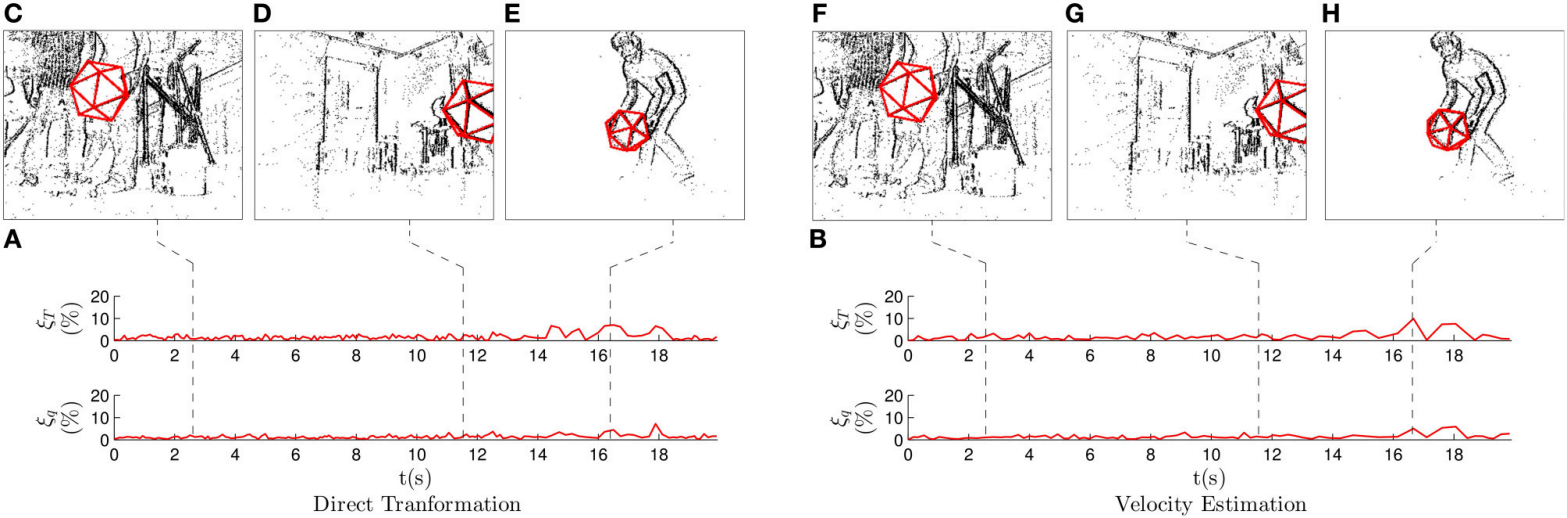
**Results for the third experiment, where we recorded a hand-held icosahedron while the camera moved to follow it**. Snapshots show the state of the system at some characteristic moments. ***(*A)** Translation and rotation errors when applying the *direct transformation* strategy. The errors remain low, always below 10%. ***(*B)** Translation and rotation errors when applying the *velocity estimation* strategy. ***(*C–H)** Snapshots showing the state of the system. We observe a large number of events produced by the egomotion of the camera. However, pose estimation is correctly performed.

Figure 9A shows the evolution of the errors when applying the *direct transformation* strategy, with λ_*T*_ = 0.4, λ_θ_ = 0.2, *N* = 5 and *m* = 4 (we do not show ***T*** or ϕ***r*** in order to lighten the figures). We verify that the estimation errors remain low for the whole recording, always below 10%.

Figure 9C shows the state of the system while the camera is moving: as we can see, the number of events is much higher in this case, as a result of the camera not being static. Consequently, most of these events are not generated by the tracked object, but rather by other visible edges in the scene. However, we verify that pose estimation is correctly performed, since the errors remain low and the projection of the estimation is coincidental with the position of the events. In Figure 9D we can see how pose estimation is performed even when a fraction of the icosahedron has left the field of view of the camera. Figure 9E shows one of the instants in which the errors reach their highest values. This happens when the object is at its furthest position from the camera, and thus when we are less precise (a pixel will represent a larger 3D distance when points are further away from the camera). However, even at this moment errors remain below 10% and the projection of the estimation is almost coincidental with the events. We conclude that pose estimation is correctly performed even in this complex scenario, providing the following mean values for the estimation errors: $\bar{\xi_{T}}=1.65\%$ and $\bar{\xi_{q}}=1.29\%$.

Figure 9B shows the evolution of the errors for the whole experiment when applying the *velocity estimation* strategy, with λ_ν_ = 0.2, λ_ω_ = 0.4, *N* = 10 and *m* = 8. The obtained results are very similar to those of the *direct transformation*, and the mean errors take the following values: $\bar{\xi_{T}}=1.72\%$ and $\bar{\xi_{q}}=1.35\%$. Figures 9F–H display the output of the system at the same instants as for the previous strategy, showing very similar results. As in the first experiment, we verify that in the case of simple objects without ambiguous positions, keeping an estimation of the velocity does not provide any advantage.

This experiment shows how the method can perform pose estimation even in complex scenarios, by simply adding some additional criteria for the matching of events. The corresponding results are displayed in Supplementary Video 3. In this case, the video depicts the whole 3D scene, showing the motion of both the camera and the tracked object.

### 3.4. Fast spinning object

**Figure 10:**
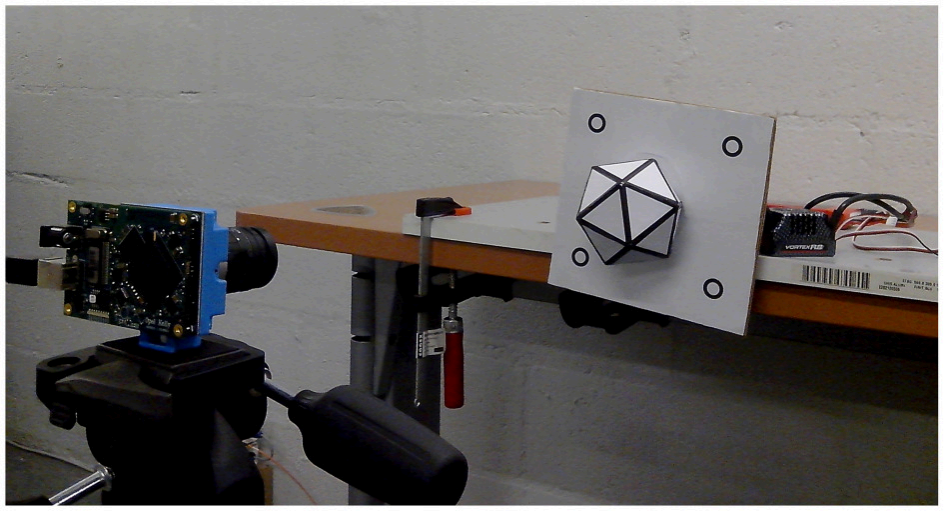
**Experimental set-up for the fast spinning experiment**. An icosahedron is attached to a brushless motor and recorded by the event-based camera. The four dots on the plane are used for ground truth.

In order to test the accuracy of the algorithm with fast moving objects, we attached the icosahedron to an electric brushless motor and recorded it at increasing angular speeds. As shown in Figure 10, the icosahedron is mounted on a plane with four dots, used for ground truth. These four points are tracked using the *Spring-Linked Tracker Set* described in Reverter Valeiras et al. (2015).

Through electronic control of the motor, we created four sections during which the angular speed is approximately constant. From the obtained ground truth, we can estimate the corresponding velocities **ν** and ω. We obtain a maximum angular speed of 26.4 rps.

The estimation errors are, for an experimentally selected optimal set of parameters: $\bar{\xi_{T}}=1.06\%$, $\bar{\xi_{q}}=3.95\%$ for the *direct transformation* strategy, and $\bar{\xi_{T}}=1.16\%$, $\bar{\xi_{q}}=4.71\%$ for the *velocity estimation* strategy. The *velocity estimation* strategy provides in this case provides less accurate results. This is due to the large angular acceleration (even if the angular speed remains approximately constant, the object is not perfectly aligned with the axis of the motor, and thus the rotation axis changes constantly). However, the mean values for the errors are low enough to conclude that, in general, the pose is correctly estimated even for objects moving at high velocity.

The results produced by the algorithm when tracking the fast spinning icosahedron are shown in Supplementary Video 4. In the video, we gradually slow down the display, allowing us to appreciate the true motion of the icosahedron. Let us note that this video was created at 25 fps, causing what is known as the *wagon-wheel illusion*. Thus, until the video is played 8 times slower than real time we do not appreciate the true direction of the rotation.

### 3.5. Degraded temporal resolution

**Figure 11:**
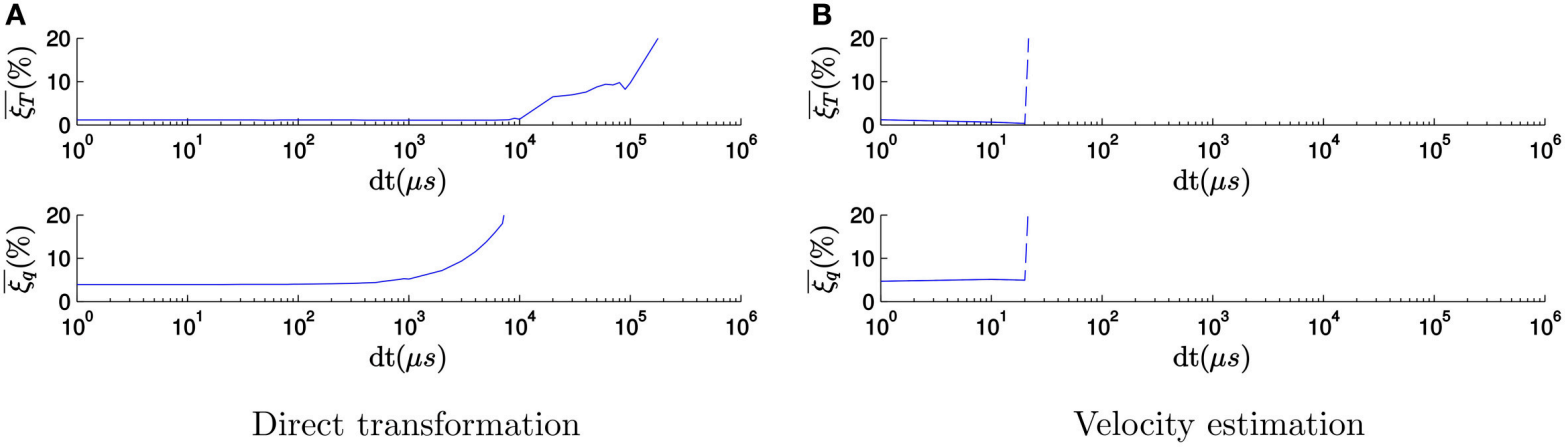
**Evolution of the errors with the size of the binning window ***dt*** (in μ s), when tracking the fast spinning icosahedron applying the ***direct transformation*** strategy**. As the time resolution is degraded, the errors start growing, until the tracker gets completely lost for values above 10 ms. **(A)** Evolution of the errors when applying the direct transformation strategy. **(B)** Evolution of the errors when applying the velocity estimation strategy.

In order to test the impact of the acquisition rate and to emphasize the importance of the high temporal resolution on the accuracy of our algorithm, we repeated the previous experiment progressively degrading the temporal resolution of recorded events. To degrade the temporal resolution, we select all the events occurring within a given time window of size *dt* and assign the same timestamp to all of them. If several events occur at the same spatial location inside of this time window, we only keep a single one. We also shuffle the events randomly, since the order of the events contains implicit high temporal resolution information. Figure 11A shows, in semi-logarithmic scale, the evolution of both the mean relative translation error and the mean relative rotation error with the size of the time window when tracking the fast spinning icosahedron applying the *direct transformation* strategy, with a fixed set of tuning parameters taken from the previous step. We only plot errors between 0 and 20%, since we consider the estimation to be unsuccessful for errors above 20%. The errors remain approximately stable until the time window reaches 1 ms. This can be explained because the small motion assumption is experimentally satisfied for time windows of 1 ms for the typical velocity in this recording. From this point on the errors start growing, until the tracker gets completely lost for values above 10 ms.

When applying the *velocity estimation* strategy, if the temporal resolution is degraded we lose track of the object very rapidly. This happens because the estimation of the velocity is based on the precise timing between events. When this information is lost, Δ*t* in Equations (17) and (18) becomes 0, which makes the estimated velocity infinite. As a result, the tracking gets lost. For the current set of parameters, this occurs for values of *dt* above 30 μ s, as one can see in Figure 11B.

We conclude from this experiment that the high temporal resolution of the neuromorphic camera output is a key feature to the successful performance of the 3D pose estimation algorithm. Beyond 10 ms pose estimation becomes a difficult problem. 10 ms is already smaller than the frame interval used by conventional computer vision algorithms.

### 3.6. Computation time

The presented experiments were carried out using a conventional laptop, equipped with an Intel Core i7 processor and running Debian Linux, while the algorithm was implemented in C++. The code was not parallelized, and just one core was used.

**Figure 12:**
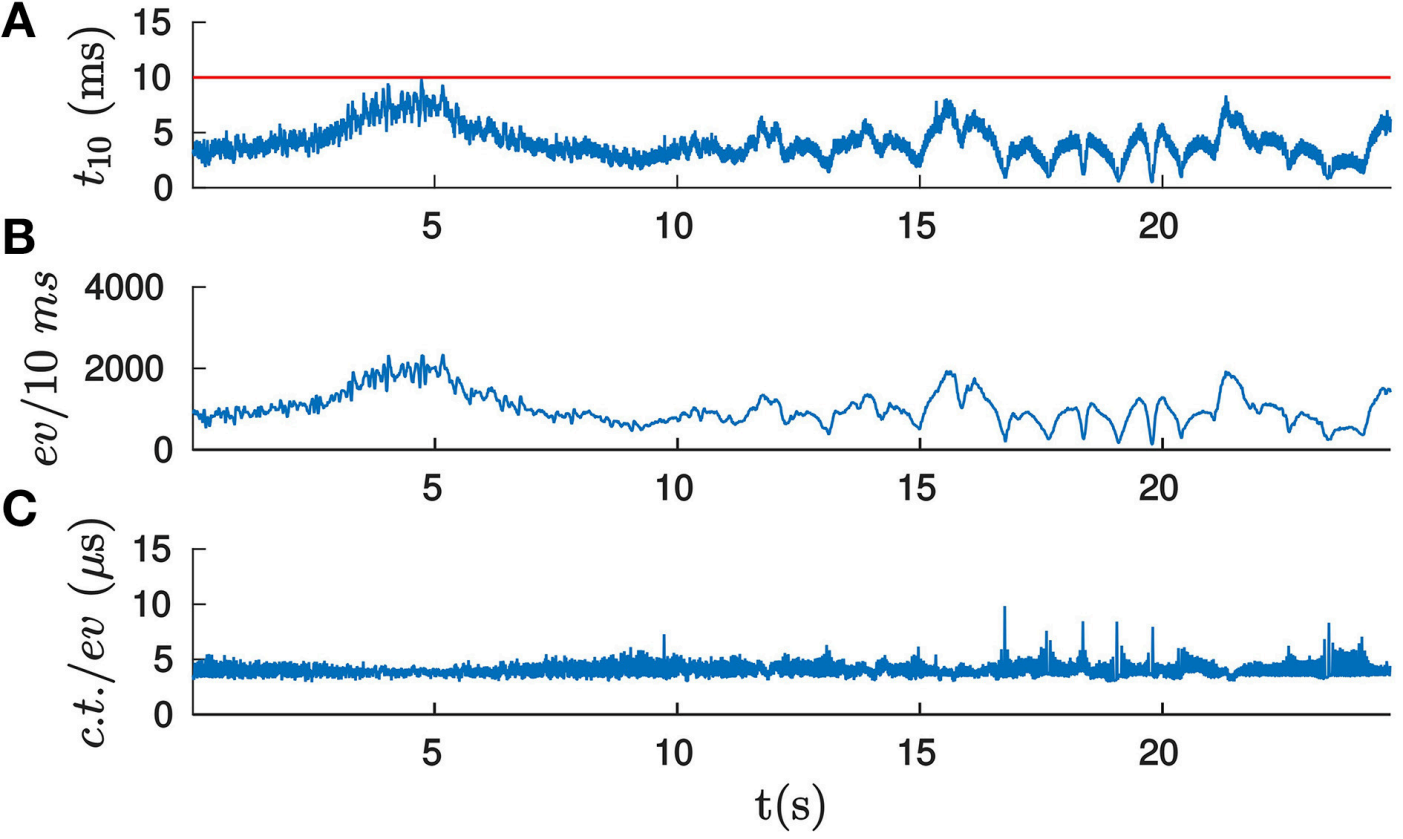
**(A)** Computational time (in ms) required for processing 10 ms of events when computing the pose of the icosahedron, applying the *velocity estimation* strategy with the experimentally selected optimal set of parameters. If *t*_10_ is below 10 ms (indicated here by the horizontal line), then the computation is carried out in real-time. ***(*B)** Number of incoming events per 10 ms. As we can observe, the number of events and *t*_10_ has a similar form, suggesting that the computational time per event remains approximately constant. ***(*C)** Computational time required per event (in μs). As we can see, it remains almost constant for the whole experiment, its mean value being equal to 5.11 μ s.

Let *t*_10_ be the time required to process 10 ms of events (10 ms is a pure technical choice, due to the software architecture used). Consequently, if *t*_10_ is below 10 ms, we consider the computation to be performed in real-time. Figure 12A shows the computational time required for processing the icosahedron sequence, when applying the *velocity estimation* strategy with the experimentally selected optimal set of parameters. The horizontal line indicates the real-time threshold. This threshold is never exceeded by the implementation. We will characterize the performance of the algorithm by the mean value of the computational time $\bar{t_{10}}$, equal in this case to 4.99 ms.

The variability in *t*_10_ can be explained by the variations in the rate of incoming events. Figure 12B shows the number of incoming events per 10 ms for the corresponding recording. This curve has a similar shape to the *t*_10_ one, suggesting that the computational time per event is stable through the whole recording. Dividing *t*_10_ by the number of incoming events gives us the computational time per event, as shown in Figure 12C. We verify that it remains approximately constant. Its mean value is equal to 5.11 μ s, which imposes a maximum rate of events that can be treated in real time equal to 195 events/ms.

**Figure 13:**
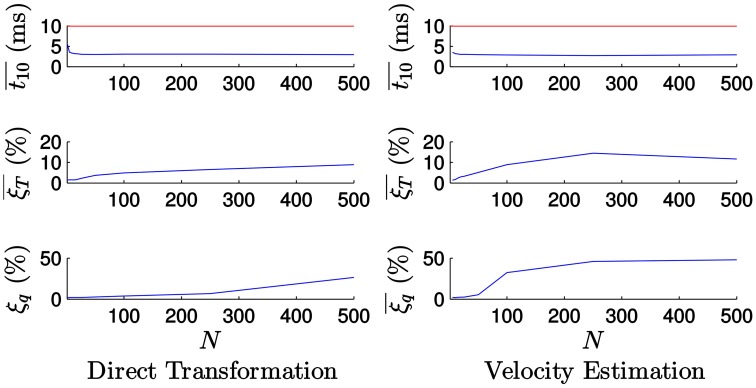
**Evolution of the errors and the computational time with the value of ***N*** when tracking the icosahedron**. $\bar{t_{10}}$ is the mean computational time required for processing 10 ms of events. **Left**: Results when applying the *direct transformation* strategy. The computational time decreases with the value of *N* until it reaches a plateau, while the errors increases with the value of *N*. **Right**: Results when applying the *velocity estimation* strategy. The computational time decreases and the errors increases with the value of *N*.

We next study the effect of the parameter *N* in the computational time and the estimation errors. Figure 13 shows the corresponding results when tracking the icosahedron, with *N* taking values between 1 and 500. Here, the mean computational time $\bar{t_{10}}$ is obtained as the mean value for 10 simulations.

**Figure 14:**
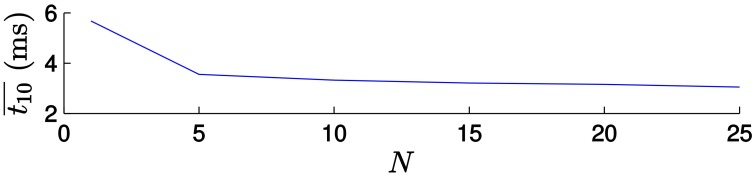
**Evolution of the computational time $\bar{t_{10}}$ for the first values of *N***. We can clearly see how the computational time is largely reduced for small values of *N*, but then it reaches a plateau.

Figure 13 (left) shows the results when applying the *direct transformation* strategy. For small values of *N* the computational time decreases as the value of *N* increases, but then it reaches a plateau. In order to illustrate this behavior more clearly, let us examine the evolution of $\bar{t_{10}}$ for small values of *N* (between 1 and 25), as shown in Figure 14. In this cases, the computational time is largely reduced for the first values of *N*, but then it is almost insensitive to its value. This can be explained if we consider that the computational time consists of the time required to update the estimation with each incoming event—which does not vary with the value of *N*—and the time required for actually applying the transformation to the model, which is a computationally expensive process, only applied every *N* events. For small values of *N*, the relative importance of the time required for transforming the model is large. Consequently, increasing the value of *N* will have a strong impact on the computational time. As *N* gets larger, the relative importance of this process is smaller, and increasing *N* will have a weaker effect.

We verify as well that the tracking errors grow with *N*. This occurs because for large values of *N* the small motion assumption is not true anymore, and thus the algorithm fails to yield correct results. In other words, when we accumulate too many events we are losing the high temporal resolution of the data, and the accuracy of the pose estimation will therefore degrade.

Figure 13 (right) shows the results when applying the *velocity estimation* strategy. For values of *N* below 5 the method is unstable, losing track of the object and producing errors that tend to infinity. As in the case of the degraded temporal resolution, this happens because Δ*t* in Equations (17) and (18) can be equal to 0. Above this value, the computational time soon reaches its plateau and is not too affected by the value of *N*. We verify that the computational time required for applying the *direct transformation* and the *velocity estimation* strategies are very similar. The errors grow with the value of *N* as well, but in this case they do it faster. We conclude that we need a higher temporal resolution to correctly estimate the velocity of the object.

**Figure 15:**
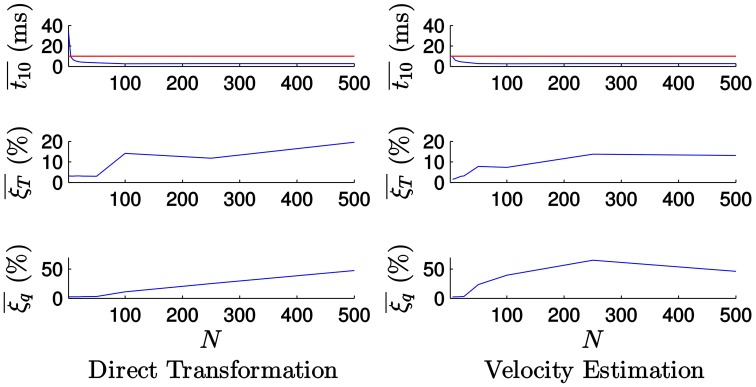
**Evolution of the errors and the computational time with the value of ***N*** when tracking the house**. For small values of *N* we cannot guarantee real-time performance. However, slightly increasing the value of *N* the computational time is reduced, with small effect on the accuracy.

Figure 15 shows the evolution of the computational time and the tracking errors with the value of *N* when tracking the house. Figure 15 (left) shows the corresponding results when applying the *direct transformation* strategy: as one can see, the results are similar to the ones obtained when tracking the icosahedron, but the computational time is much higher in this case. This is mainly due to the higher complexity of the hidden line removal algorithm. As we can see, for small values of *N* we cannot guarantee real-time performance. However, as the value of *N* grows, the computational time is reduced.

We verify as well that the errors grow with the value of *N*. Nevertheless, for small values of *N* there is a plateau in which they are very slightly affected by its value. For example, for *N* = 25 we get $\bar{\xi_{T}}=3.129\%$ and $\bar{\xi_{q}}=2.776\%$, while the mean computational time is $\bar{t_{10}}$ = 4.123 ms. Therefore, we get low values for the estimation errors while keeping the computational time below the real-time threshold. We conclude that it is possible to guarantee real-time performance even for this more complex object by slightly increasing the value of *N*, with small effect on the accuracy. The same considerations apply in the case of the *velocity estimation* strategy, shown in Figure 15 (right).

## 4. Discussion

This paper introduces a new method for 3D pose estimation from the output of a neuromorphic event based camera. To our knowledge, this is the first 3D pose estimation algorithm developed using this technology. The method is truly event-driven, as every incoming event updates the estimation of the pose. The transformation applied with each event is intuitively simple and uses the distance to the line of sight of pixels.

We showed that the method is able to estimate and track 3D moving objects at high accuracy and low computational costs by exploiting the high temporal resolution of the event-based sensor. Depending on the recording and the method chosen, we get translation errors ranging from 1.06 to 3.12% and rotation errors from 1.29 to 4.71%. These values are reasonably low for us to conclude that pose estimation is correctly performed.

We have also shown that when the temporal resolution of the events is degraded to simulate frame based conditions, a point is reached after which the pose cannot be accurately estimated. In the studied recording, this happens when the temporal resolution is 10 ms in the case of the *direct estimation* strategy, or 30 μs when the *velocity estimation* strategy is applied. We conclude that the high temporal resolution of the neuromorphic camera is a key feature to the accuracy of our algorithm.

Compared to frame-based methods, we consider our approach to be conceptually simpler. Instead of redundantly processing all pixels, as it is usually done in the frame based approach, the event-based philosophy is to minimize the computational resources applied to each event. Once we are close to the solution, the event-based approach allows us to continuously track the correct pose, thanks to the high temporal precision of the sensor. As a canonical example, we are able to accurately estimate the pose of an object spinning at angular speeds up to 26.4 rps. To achieve equivalent accuracy with a frame-based camera, high frame rates would be required, and consequently the number of frames to process will increase.

The method can also be used in mobile scenarios by applying more robust matching algorithms relying on additional matching criteria, such as the local orientation of edges. The method is robust to partial occlusions and does not impose any limitation on the type of model that can be used. The only constraint is given by the increase in computational time associated with the complexity of the object specially in computing hidden surfaces. Other models, including parametric curves or point clouds, could be used with very small modifications to the algorithm. In the case of real-time requirements, we show that the tuning of the parameter *N* provides lower computational times with little impact on the accuracy of the pose estimation.

We have also shown how an assumption of velocity smoothness can improve pose estimation results when an expected rate of change of velocity is known for the object. This being a reasonable hypothesis, the *velocity estimation* strategy is in most cases the standard choice. The *direct transformation* strategy should be chosen when high values for the acceleration are expected.

## Author contributions

DR: Main contributor. Formalized the theory, implemented the experiments and evaluated the results. GO: Provided support for the experimental setup and participated in the experiments. SI: Co-supervisor. RB: thesis director and main instigator of the work.

## Funding

This work received financial support from the LABEX LIFESENSES [ANR-10-LABX-65] which is managed by the French state funds (ANR) within the Investissements d'Avenir program [ANR-11-IDEX-0004-02].

### Conflict of interest statement

The authors declare that the research was conducted in the absence of any commercial or financial relationships that could be construed as a potential conflict of interest.

## Footnotes

^1^All recordings and the corresponding ground truth data are publicly available at https://drive.google.com/folderview?id=0B5gzfP0R1VEFNS1PZ0xKU3F5dG8&usp=sharing.

## Appendix

In this section, we discuss the solutions to the system of equations defined in Equation (8). Let *S* be the system matrix, that has the form:

(A1) $$\begin{array}{l} S=\left(\begin{array}{c} -M_{k}^{T}M_{k}M_{k}^{T}\varepsilon_{nm}\\ -M_{k}^{T}\varepsilon_{nm}\varepsilon_{nm}^{T}\varepsilon_{nm} \end{array}\right). \end{array}$$

Next, we discuss the solutions to this system, both in the singular and in the general case.

### Singular case

The system matrix *S* is singular when *det*(*S*) = 0, where the determinant takes the following value:

(A2) $$\begin{array}{l} det\left(S\right)=-\left(M_{k}^{T}M_{k}\right)\left(\varepsilon_{nm}^{T}\varepsilon_{nm}\right)+{\left(M_{k}^{T}\varepsilon_{nm}\right)}^{2}. \end{array}$$

Developing the dot products in this equation, we get:

(A3) $$\begin{array}{l} \begin{array}{c} det\left(S\right)=0\Leftrightarrow ∥M_{k}{∥}^{2}∥\varepsilon_{nm}{∥}^{2}={\left(∥M_{k}∥∥\varepsilon_{nm}∥cos\left(\gamma \right)\right)}^{2},\\ det\left(S\right)=0\Leftrightarrow cos\left(\gamma \right)=\pm 1. \end{array} \end{array}$$

where γ is the angle between ***M***_*k*_ and ε_*nm*_.

Consequently, *S* will be singular if γ = 0 or γ = π, this is equivalent to have ***M***_*k*_ collinear to ε_*nm*_. In this case, ***B***_*k*_ is chosen between ***V***_*n*_ and ***V***_*m*_ by taking the one with smaller *Z* coordinate. We then compute α_1_ from the perpendicularity constraint between ***M***_*k*_ and (***A***_*k*_ − ***B***_*k*_), getting the following result:

(A4) $$\begin{array}{l} \alpha_{1}=\frac{B_{k}^{T}M_{k}}{M_{k}^{T}M_{k}}, \end{array}$$

and insert this value in Equation (6) to obtain ***A***_*k*_.

### General case

In the general case, *S* will be invertible. Since *S* is a 2 × 2 matrix, we can analytically precompute its inverse, saving computational power. In order to solve the system, we define the following dot products

(A5) $$\begin{array}{l} \begin{array}{c} a=M_{k}^{T}M_{k}\\ b=\varepsilon_{nm}^{T}\varepsilon_{nm}\\ c=M_{k}^{T}\varepsilon_{nm}\\ d=V_{n}^{T}M_{k}\\ e=V_{n}^{T}\varepsilon_{nm} \end{array} \end{array}$$

Thus, the inverse will have the following expression:

(A6) $$\begin{array}{l} inv\left(S\right)=\frac{1}{det\left(S\right)}\left(\begin{array}{c} b-c\\ c-a \end{array}\right), \end{array}$$

where *det*(*S*) = −*ab* + *c*^2^. This allows us to solve the system for α_1_ and α_2_. As a final observation, we need to take into account that ε_*nm*_ is a segment, which means that α_2_ has to be contained in the interval [0, 1]. Thus, the final values for α_1_ and α_2_ are:

(A7) $$\begin{array}{l} \alpha_{1}=\frac{-bd+ce}{det\left(S\right)} \end{array}$$

and

(A8) $$\begin{array}{l} \alpha_{2}=\{\begin{array}{ll} 0, & \text{ if }\;\frac{-cd+ae}{det(S)}\leq 0\\ 1, & \text{ if }\;\frac{-cd+ae}{det(S)}\geq 1\\ \frac{-cd+ae}{det(S)}, & \text{ otherwise} \end{array} \end{array}$$

Inserting these values in Equations (6) and (7) provides ***A***_*k*_ and ***B***_*k*_.
